# Enhanced CO_2_ Capture of Poly(amidoamine)-Modified Graphene Oxide Aerogels with the Addition of Carbon Nanotubes

**DOI:** 10.3390/ijms24043865

**Published:** 2023-02-15

**Authors:** Alina Iuliana Pruna, Alfonso Cárcel, Adolfo Benedito, Enrique Giménez

**Affiliations:** 1 Instituto de Tecnología de Materiales, Universitat Politècnica de València (UPV), Camino de Vera s/n, 46022 Valencia, Spain; 2 Center for Surface Science and Nanotechnology, University Politehnica of Bucharest, 316 Splaiul Independentei, 060042 Bucharest, Romania; 3 Instituto Tecnológico del Plástico (AIMPLAS), 4 Gustave Eiffel, Paterna, 46980 Valencia, Spain

**Keywords:** graphene oxide, aerogel, dendrimer, carbon nanotubes

## Abstract

Innovative dendrimer-modified graphene oxide (GO) aerogels are reported by employing generation 3.0 poly(amidoamine) (PAMAM) dendrimer and a combined synthesis approach based on the hydrothermal method and freeze-casting followed by lyophilization. The properties of modified aerogels were investigated with the dendrimer concentration and the addition of carbon nanotubes (CNTs) in varying ratios. Aerogel properties were evaluated via scanning electron microscopy (SEM), Fourier transform infrared spectroscopy (FTIR), Raman spectroscopy, and X-ray photoelectron spectroscopy (XPS). The obtained results indicated a strong correlation of the N content with the PAMAM/CNT ratio, where optimum values were revealed. The CO_2_ adsorption performance on the modified aerogels increased with the concentration of the dendrimer at an appropriate PAMAM/CNT ratio, reaching the value of 2.23 mmol g^−1^ at PAMAM/CNT ratio of 0.6/0.12 (mg mL^−1^). The reported results confirm that CNTs could be exploited to improve the functionalization/reduction degree in PAMAM-modified GO aerogels for CO_2_ capture.

## 1. Introduction

Carbon dioxide (CO_2_) is considered one of the largest drivers of global warming. To mitigate the effects of CO_2_ emissions, many materials have been studied for porous sorbents including metal–organic frameworks (MOFs), zeolites, and carbon-based and metal oxides [1,2,3]. Amongst these alternatives, three-dimensional porous networks based on graphene oxide (GO) nanomaterials have raised increased interest, thanks to the benefits in processability offered by the oxygen-containing functional groups of GO, as well as GO large surface area and low density [4].

Varying approaches to improving the sorption properties of GO-based sorbents [5] include GO oxidation degree, flake size, concentration, synthesis method, or synthesis [6,7] conditions, which are mainly directed to tailor the porosity properties. The hydrothermal method is one of the most employed given its simplicity and the low temperature needed [8]. In this regard, aspects including the temperature, duration, the use of a reducing agent, and the solution’s pH were employed to tailor the properties of the resulting hydrogel [9]. Freeze-drying methods [10] are then applied to obtain the corresponding aerogel by sublimating the solvent ice crystal and thus result in pores that impede the restacking of the GO sheets. The aerogel porosity parameters, including the size, shape, and distribution of solvent ice crystals, can be further tailored by the freeze-casting method [11]. Since the resulting pores mirror the frozen solvent crystals [12], the thermal, electrical, compressive, and sorption properties could be enhanced by adjusting the cooling rate, e.g., freeze-casting in liquid nitrogen [13].

Another typical approach to improve the properties of GO sorbents is based on exploiting the surface chemistry of GO to attach amine-containing molecules, e.g., ethylenediamine [14]. Recently, poly(amidoamine) (PAMAM) dendrimers have gained research interest for CO_2_ capture as they exhibit the advantage of a higher number of functional amine end groups that are available for cross-linking [15,16,17]. However, amongst the works on dendrimer-modified CO_2_ sorbents, only a few deal with modified GO aerogels [18].

It is known that dendrimer features such as size, shape, and amine groups vary with dendrimer generation [19]. As the number of amine groups increases exponentially with dendrimer generation, one would expect the functionalization degree with dendrimer to increase. However, the shape of the dendrimer and its concentration were reported to greatly affect the sorption properties of modified GO aerogels [18]. More specifically, CO_2_ capture was shown to decrease with dendrimer concentration and size due to steric hindrance, i.e., the functionalization with a smaller planar dendrimer molecule from generation 3.0 yielded better results than its larger spherical counterpart from generation 7.0 [20].

Often, carbon nanotubes (CNTs) have been reported in composites with GO, resulting in a synergic effect thanks to their π–π links and improved features [21]. Since aspects such as sheet stacking, porosity, and functionalization degree in modified GO composites could be tackled by adding a spacer to the assemblies, CNTs received much attention in this respect [22,23].

In this work, CNTs were employed to enhance the functionalization of GO with a generation 3.0 dendrimer. PAMAM–GO/CNT aerogels were obtained using the hydrothermal method and freeze-casting in liquid nitrogen. The effect of CNT on the properties of modified aerogels was studied with varying dendrimer/CNT concentration ratios. The presented results indicate that the functionalization degree and sorption performance could be tailored by using a proper balance between dendrimer and CNT content.

## 2. Results and Discussion

PAMAM dendrimer generation 3.0 in varying concentrations was employed to modify GO aerogels through hydrothermal synthesis. The properties of the PAMAM-modified GO aerogels were studied with the content of CNTs (namely, the concentration ratio PAMAM/CNT, mg L^−1^).

**Figure 1:**
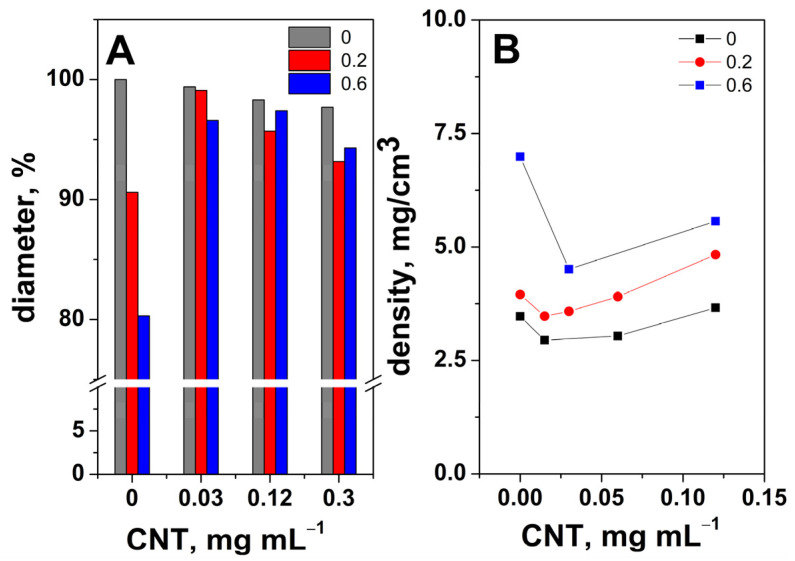
Evolution of diameter (**A**) and density (**B**) for the PAMAM-modified (mg mL^−1^) aerogels with CNT content.

First, the dimensions of the obtained aerogel were studied with synthesis conditions. No significant indication of the vertical contraction of the monolith was observed with the dendrimer or CNT content, due to the short hydrothermal synthesis duration of only 4 h. On the other hand, the diameter of hydrogel was affected due to the confined space, as shown in Figure 1.

Figure 1A depicts the diameter evolution of dendrimer-modified aerogel with the CNT content, where the reference (100%) is represented by the aerogel obtained in the absence of either PAMAM or CNTs, that is, a PAMAM/CNT ratio of 0/0. The addition of CNTs to pure GO aerogels slightly decreased the diameter, which was attributed to the separating role of CNTs, thus exposing them to reduction. The functionalization with the dendrimer of the GO aerogels resulted in a diameter decrease, especially at a higher concentration (0.6 mg mL^−1^), which was attributed to the GO sheet stacking induced by an increased degree of functionalization. However, the addition of CNTs to the PAMAM-modified GO aerogel managed to increase the diameter for both given PAMAM concentrations close to the value obtained in the absence of PAMAM, which indicates a synergetic effect between PAMAM and CNTs toward tailoring the functionalization process. A similar trend was exhibited for both PAMAM concentrations upon adding the CNTs, with a maximum of 99% and 97% diameter being recorded at similar PAMAM/CNT ratios. Upon the addition of CNTs, the diameter of the PAMAM-modified aerogels increased up to a maximum, which can be explained by CNTs separating the GO sheets. The decrease recorded afterward indicated the deficient separation of GO sheets attributed to CNT aggregation. Thus, the optimum CNT content was recorded as 0.03 and 0.12 mg mL^−1^ when the PAMAM concentration was 0.2 and 0.6 mg mL^−1^, respectively.

The density evolution for the aerogels modified with PAMAM and CNTs is depicted in Figure 1B. The density ranged between 2.5 and 7 mg cm^−3^, in agreement with similar aerogels [18]. Adding PAMAM molecules resulted in an increase in the modified aerogel density, reaching a maximum of 7 mg cm^−3^ for the higher content of PAMAM, which was attributed to the higher degree of functionalization. Upon the addition of CNTs, aerogel density decreased, reaching a minimum, and this was attributed to the spacer effect of CNTs that increased the reduction in GO sheets.

By comparing the diameter evolution with the one for density, it can be observed that the same PAMAM/CNT concentration ratios were recorded for minimum density and maximum diameter, which was indicative of the synergy between the CNTs and PAMAM in also tailoring the aerogel porosity. As explained in the following sections, the PAMAM-modified aerogels that were investigated corresponded to the optimum PAMAM/CNT ratios of 0.2/0.03 and 0.6/0.12.

**Figure 2:**
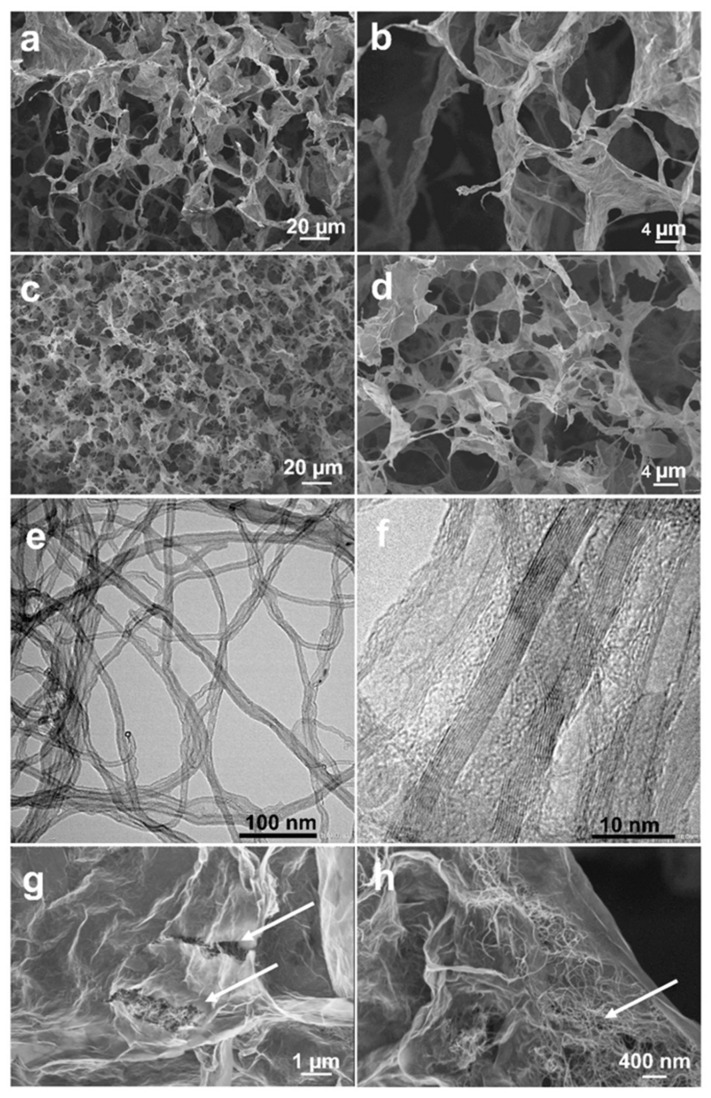
SEM images of increasing magnification (from left to right) of: pure GO aerogel (**a**,**b**), PAMAM (0.2 mg mL^−1^) modified GO aerogel (**c**,**d**), CNTs (**e**,**f**), and PAMAM/CNT (0.2/0.06 mg mL^−1^) modified GO aerogel (**g**,**h**).

The SEM images in Figure 2 depict the typical morphology of the aerogels upon PAMAM/CNT modification. For exemplification, the case of 0.2 mg mL^−1^ PAMAM-modified aerogel is presented. Porosity and GO sheet stacking were similar to those observed in other reports [18]. One can see that the non-modified aerogel in Figure 2a,b presents a rather non-homogenous distribution of pores with irregular dimensions, and the contrast of GO sheets indicated a certain stacking. The PAMAM-modified aerogel depicted in Figure 2c,d shows the improved distribution of smaller pores, and the GO sheets appear to exhibit less stacking given their increased transparency, which is attributed to functionalization with a high number of amine groups in PAMAM molecules. Furthermore, the CNTs depicted in Figure 2e,f present a high length of several microns and a large diameter of up to 15 nm, which corresponds to around 15 walls of CNTs. The successful incorporation of CNTs between PAMAM-modified GO sheets is confirmed with the higher magnification images in Figure 2g,h, as indicated by the arrows. An aggregate of CNTs is shown, given the use of higher CNT concentration necessary for their identification at higher magnification. The aggregation of CNTs was in line with the evolution of the aerogel diameter.

**Figure 3:**
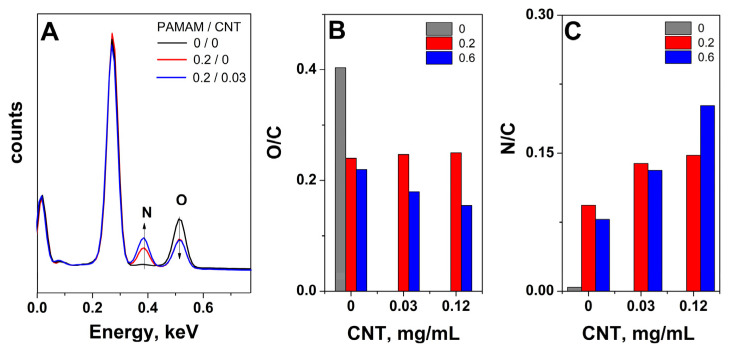
Typical EDS spectra of PAMAM/CNT-modified aerogels (mg mL^−1^) (**A**); evolution of O/C at. ratio (**B**) and N/C at. ratio (**C**) for the PAMAM-modified aerogels (mg mL^−1^) with CNT content.

The functionalization with PAMAM was further studied with EDS analysis. The typical spectra depicted in Figure 3A show that the oxygen content decreased simultaneously with an increase in nitrogen content upon functionalization with the dendrimer, which indicates that functionalization takes place simultaneously with the reduction in GO sheets [18,24]. On the other hand, the presence of CNTs appeared to improve the functionalization degree with PAMAM, as the nitrogen content further increased.

To better understand the functionalization process with dendrimers in the presence of CNTs, the evolution of O/C and N/C atomic ratios for the aerogels with varying PAMAM/CNT concentration ratios is presented in Figure 3B,C. A clear reduction in GO sheets upon modification with the dendrimer was observed with the decrease in the O/C ratio, as shown in Figure 3B. Regarding the addition of CNT to dendrimer–GO aerogels, the low-concentration dendrimer-modified aerogels did not exhibit further reduction upon the addition of CNTs; however, the effect was clear in the case of higher dendrimer concentration, as the O/C still decreased with an increase in the CNT content up to the optimum level of 0.12, which could be attributed to an improvement in the functionalization degree. The evolution of the N/C ratio was in line with the O/C content, that is, functionalization with PAMAM molecules was confirmed by the N content of the dendrimer-modified aerogels, slightly lower at higher PAMAM concentration due to the steric hindrance of functionalization [18]. A further increase in the N/C ratio with CNT content indicated a better functionalization reaction between the amine groups of the dendrimers and the oxygen groups on the GO sheets, which was attributed to CNTs’ role as spacers. Moreover, by adding the corresponding optimum CNT content, the N/C ratio increases 2.5 times for 0.6 mg mL^-1^ PAMAM-modified aerogel while it increases only 1.5 times for 0.3 mg mL^-1^ PAMAM-modified aerogel. These results confirm that the optimum content of modifiers needs to be considered to improve aerogel properties, in agreement with the density and diameter results.

**Figure 4:**
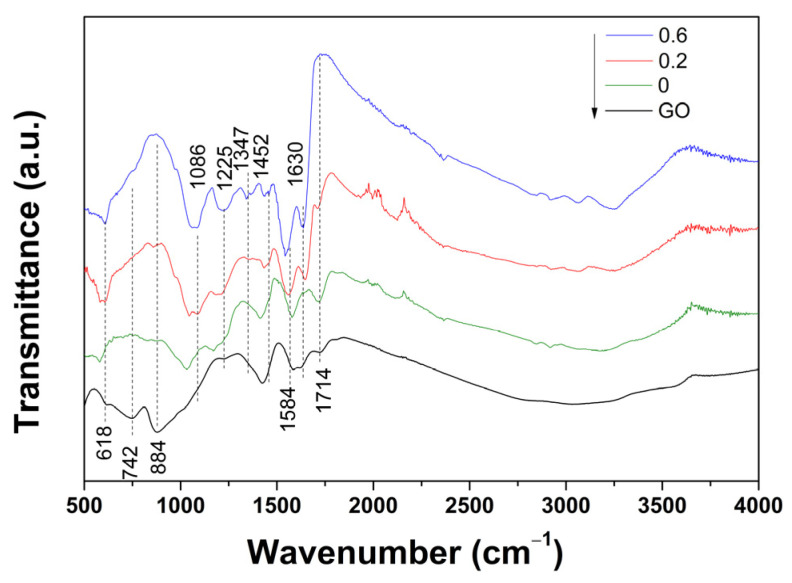
FTIR spectra of GO nanomaterials and modified GO aerogels with PAMAM concentration (mg mL^−1^).

The modification with dendrimer amine groups was monitored using FTIR analysis, as shown in Figure 4, depicting the evolution of the typical spectra of GO nanomaterial upon the incorporation of varying content of PAMAM to obtain the corresponding modified GO aerogels. The GO nanomaterial exhibited typical FTIR bands located at 1614 cm^−1^ attributed to the C=C stretching in aromatic rings and a wide band centered at 3000 cm^−1^ attributed to C–OH due to the intercalated water molecules. The various functional groups of GO were identified by the presence of bands located at 742 and 884 cm^−1^, attributed to carboxyl COOH and ketones C=O, respectively, and 1049 cm^−1^ attributed to C–O, 1218 cm^−1^ attributed to C–O–C, 1424 cm^−1^ attributed to OH and C=O, 1584 cm^−1^ attributed to C=O, and 1714 cm^−1^ attributed to COOH groups [25]. Upon the hydrothermal reduction for the synthesis of the GO aerogel, the spectra showed fewer intercalated water molecules, and while the bands located at 742 and 884 cm^−1^ disappeared, the one at 1424 cm^−1^ decreased in intensity, and the one at 1714 cm^−1^ became more intense. All these changes highlight the removal and conversion of oxygen functional groups in GO during the formation of the corresponding aerogel [24,25]. Upon the addition of the dendrimer for the synthesis of the modified aerogels, new peaks related to amides and corresponding to the coupling of the C–N stretching vibration emerged at 1549, 1452, 1347, 1225, and 1086 cm^−1^, together with another vibration band at 1630 cm^−1^ attributed to the amide C=O stretching vibration mode corresponding to NH_2_ deformation [20,26,27,28]. The appearance of the new bands confirmed the incorporation of PAMAM into the modified GO aerogels by cross-linking between the carboxylic groups in GO and amine groups in PAMAM dendrimers [20,29,30,31]. The spectral features of the aerogel modified with the increased addition of the dendrimer confirmed these findings, as the above-mentioned band modifications were more evident (i.e., more intense peaks emerged, while some peaks of GO (the band at 1714 cm^−1^) completely disappeared).

**Figure 5:**
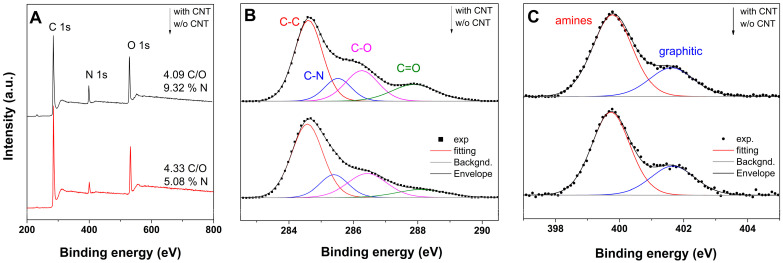
XPS survey scan (**A**); deconvoluted C 1s (**B**) and O 1s spectra (**C**) of PAMAM-modified (0.2 mg mL^−1^) aerogel in absence and presence of 0.03 mg mL^−1^ CNTs.

XPS was employed to characterize the evolution of the oxygen-containing groups of PAMAM-modified aerogels with CNT addition. For exemplification, Figure 5 depicts the XPS survey spectra, including high-resolution C 1s and N 1s spectra of the aerogel modified with the 0.2 mg mL^−1^ PAMAM dendrimer without and with 0.03 mg mL^−1^ CNTs. The survey spectra in Figure 5a show N 1s peaks besides the typical C 1s and O 1s peaks, which confirmed the incorporation of dendrimer into the aerogel network. The quantitative analysis indicated that the aerogel obtained in the absence of CNTs exhibited a C/O atomic ratio of 4.33 and 5.08 at.% N content, while the aerogel obtained in the presence of CNTs showed a decreased C/O ratio and increased N content. This revealed that the GO was further reduced with the enhanced incorporation of the dendrimer [32,33]. Upon the addition of CNTs, the C/N ratio decreased from 15.18 to half this amount, which supported the evidence for the role of CNTs as spacers for improving dendrimer incorporation.

To gain insight into the evolution of functional groups, the deconvolution of both C 1s and N 1s was analyzed. The deconvoluted C 1 s spectra of the modified aerogel, shown in Figure 5B, exhibited three typical peaks centered at 284.6, 285.5, and 288.2 eV, corresponding to the C-C/C=C group (unoxidized skeletal carbon), the C-OH/C-O-C group, and the COOH group, with a corresponding peak area ratio of 1:0.28:0.17 [34]. Upon the addition of CNTs, a peak centered at 287.8 eV attributed to C=O appeared at the expense of the COOH peak, resulting in a peak area ratio of 1:0.27:0.33. The improved incorporation of the dendrimer with CNTs was observed with a new peak related to the C-N centered at 286.3 eV and a peak area ratio (relative to C-C/C=C one) increasing from 0.41 to 0.44 for the aerogel obtained in the absence and presence of CNTs, respectively. On the other hand, the deconvoluted N 1s spectra, shown in Figure 5C, exhibited two peaks centered at 399.8 and 401.6 eV that were attributed to amines and graphitic N content [35] and exhibited a similar peak area ratio. The varying evolution of peak area ratios in the C 1s spectra indicated that functionalization with the dendrimer improved at the expense of oxygen-containing groups, such as carboxyl ones, and this finding is in good agreement with FTIR results.

**Figure 6:**
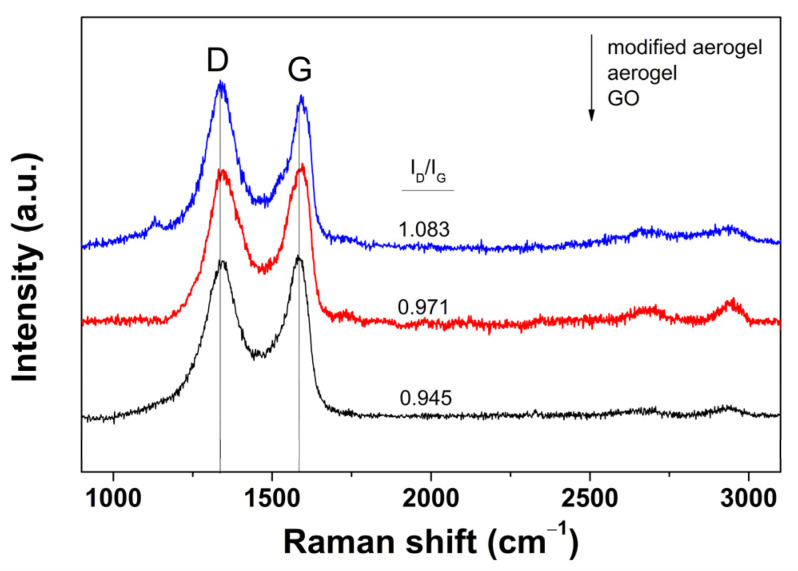
Raman spectra of initial GO dispersion and aerogels before and after dendrimer modification.

Figure 6 presents the typical Raman spectra of the initial GO dispersion and the aerogels before and after dendrimer modification (exemplification for PAMAM 0.2 mg mL^−1^). The two major bands attributed to lattice disorder (D) and originating from the graphene lattice (G) located at about 1335 and 1580 cm^−1^, respectively, were clearly observed [18]. By employing the peak intensity ratio (ID/IG) as a measure of the disorder level, one can observe the reduction in GO during the hydrothermal synthesis of the aerogel by a ratio increase from 0.945 for the initial GO dispersion to 0.971 for the aerogel. Functionalization was evidenced by a further ratio increase to 1.083 for the modified aerogel due to the grafting of the dendrimer molecules. Another indication for the modification of the graphene network in the aerogels was the shift in the G band from 1580 cm^−1^ for GO dispersion to 1595 and 1595 cm^−1^ for the aerogel before and after dendrimer modification [18].

**Figure 7:**
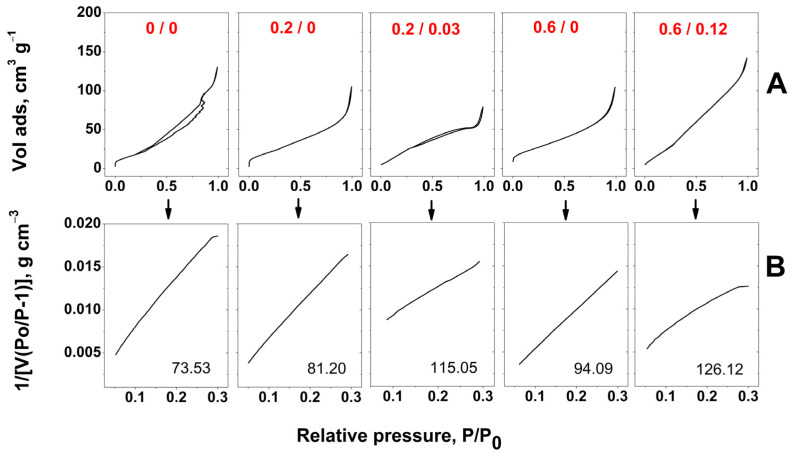
Full nitrogen adsorption isotherms (**A**) and corresponding BET plots including BET surface area (m^2^ g^−1^) (**B**) on the aerogels modified with PAMAM/CNTs (mg mL^−1^).

The CNT effect on the BET surface area of aerogels was further analyzed. Figure 7 depicts the nitrogen adsorption isotherms and the corresponding BET surface area plots for the aerogels before and after modification with PAMAM/CNT (mg mL^−1^). The BET surface area (m^2^ g^−1^) obtained from the plots is shown in the figures. One can observe that the non-modified aerogel had a BET surface area of approx. 74 m^2^ g^−1^, in line with similar aerogels [18]. The BET surface area increased with PAMAM concentration due to the increased functionalization degree [18], and further addition of CNTs to the optimum PAMAM/CNT concentration ratio, namely 0.2/0.03 and 0.6/0.12, facilitated a further increase in the BET surface area to 115 and 126 m^2^ g^−1^, respectively. These results highlight the functionalization process with PAMAM and the spacer role of CNTs.

**Figure 8:**
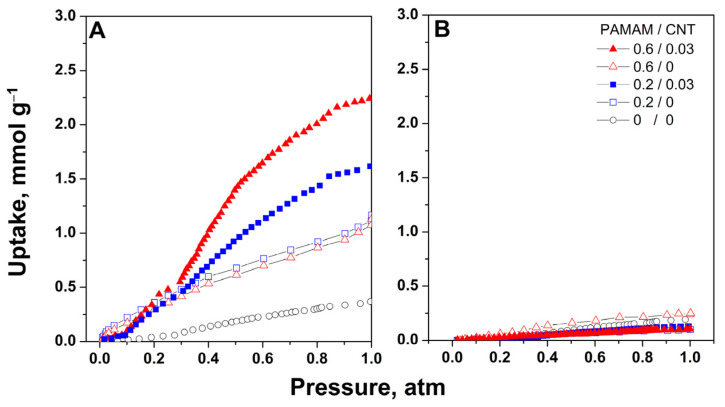
CO_2_ (**A**) and N_2_ (**B**) adsorption isotherms on GO aerogels before and after modification with PAMAM dendrimer and CNT (mg mL^−1^) addition at 25 °C.

Figure 8 presents the adsorption isotherms for both CO_2_ and N_2_ at 25 °C on the modified GO aerogels as a function of PAMAM dendrimer and CNT concentration. The improvement in the GO aerogel upon dendrimer modification was clearly observed, as the CO_2_ uptake increased from 0.38 to 1.17 mmol g^−1^. As shown in Figure 8A, the increase in dendrimer concentration did not result in a further improvement, in agreement with reported research, which is due to the spatial hindering effect on functionalization [18]. However, upon the addition of CNTs in the optimal range, as observed previously, for both cases of PAMAM concentrations (PAMAM/CNT of 0.2/0.03 or 0.6/0.13), the CO_2_ uptake markedly increased, with the highest value corresponding to the PAMAM/CNT at a higher concentration.

These results can be explained by improved adsorption due to the improved functionalization of amines achieved by using CNTs as spacers. Moreover, the shape of the isotherm changed to a sigmoidal shape with low adsorption at lower pressure and a steep increase at higher pressure, which could be due to pore filling [36], after which both aerogels modified with the dendrimer and CNTs seemed to reach a plateau at a higher pressure level. However, the aerogel with higher dendrimer concentration and corresponding optimal CNT addition recorded a higher uptake at 1 atm, which was attributed to the existence of increased porosity in the aerogel, in agreement with previous results and the reported literature on similar sorbents [37,38,39]. On the other hand, the N_2_ adsorption isotherms revealed low uptakes for all cases, which suggested the good selectivity of the obtained materials. The low uptake for N_2_ could be attributed to the availability of pores for its adsorption; especially when GO is modified with dendrimers, the introduction of the molecule might act as a shield layer, impeding the adsorption of N_2_ [40]. Consequently, the developed materials could be considered an alternative for preventing CO_2_ emissions, when an optimum balance of these components is employed.

## 3. Materials and Methods

An aqueous GO slurry (monolayer content > 95%) was supplied by Graphenea (Donostia, Spain), while CNTs (NC7000 series, multiwall, average diameter 9.5 nm, average length 1.5 μm) were supplied by Nanocyl (Sambreville, Belgium). Polyvinylpyrrolidone (PVP–K90, molecular biology grade) was supplied by Scharlab (Barcelona, Spain), and the PAMAM dendrimer (ethylenediamine core, generation 3.0, solution 20 wt.% in methanol) was supplied by Sigma-Aldrich (Madrid, Spain). The rest of the reagents were supplied by Alfa Aesar (Madrid, Spain).

A 2 mg mL^−1^ GO dispersion was obtained by adding an adequate amount of GO slurry to distilled water and through homogenization in an ultrasonic bath for 1 h. A 3 mg mL^−1^ ethanolic CNT dispersion was obtained by adding CNTs to ethanol together with PVP as a dispersing agent in a 1.5:1 CNT: PVP *w*/*w* ratio followed by ultrasound treatment for 1 h.

A varying amount of dendrimer was employed for functionalization in the absence and presence of CNTs. For this purpose, the corresponding mixtures of GO and CNT solutions were first mixed, and then the dendrimer was added and subjected to further sonication. The final mixture was transferred to a sealed reactor and heated to 140 °C for 4 h in an oven. The resulting black hydrogels were then freeze-cast in liquid nitrogen. Lyophilization with a Lyomi Cron freeze dryer (Coolvacuum Technologies, Barcelona, Spain) was further applied for 3 days to obtain the corresponding aerogels.

The apparent density of the aerogels was estimated in triplicate measurements from the mass of the aerogels and their volume, employing a caliper of 0.05 mm accuracy. Morphological analysis and qualitative composition were performed by using a Gemini scanning electron microscope (SEM) connected to an energy-dispersive analyzer (Zeiss Microscopy, Oberkochen, Germany, 1.50 kV). The reduction and functionalization processes were studied via Raman spectroscopy with an Xplora spectrometer (Horiba, Villeneuve d’Ascq, France, 532 nm laser), Fourier transform infrared spectroscopy (FTIR) was performed by using an FT/IR-6200 (Jasco, Madrid, Spain) spectrometer in the ATR mode and via X-ray photoelectron spectroscopy (XPS) on a VG-Microtech Multilab 3000 spectrometer (Thermo Fisher Scientific Inc., Waltham, MA, USA). The specific surface areas of the aerogels were determined using the Brunauer–Emmet–Teller (BET) method from nitrogen adsorption isotherms recorded at 77 K by using an ASAP 2420 analyzer (Micromeritics, Norcross, GA, USA). The sorption properties were evaluated with CO_2_ and N_2_ adsorption isotherms at 298 K, up to 1 bar on an ASAP 2420 analyzer (Micromeritics). Prior to the measurement, the samples were outgassed under vacuum at 80 °C for 24 h to avoid a further reduction in the GO aerogels.

## 4. Conclusions

Novel aerogels were obtained by modifying GO–aerogels with generation 3.0 PAMAM dendrimer molecules through a combined hydrothermal route and freeze-casting approach. Liquid nitrogen was applied for freeze-casting to leave the structure of hydrogel intact upon freeze-drying and induce an increased pore homogeneity in the aerogel due to the extremely fast cooling rate. Furthermore, the modification with the dendrimer was also studied for a GO–CNT mixture.

The properties of the modified aerogels were analyzed with dendrimer concentration and CNT addition. The results indicated that a proper balance in the PAMAM/CNT concentration ratio must be considered to achieve the best aerogel properties, namely, density, functionalization degree, or sorption. For the case of low PAMAM concentration such as 0.2 mg mL^−1^, an optimum content of CNTs was found at 0.03 mg mL^−1^, while at higher dendrimer concentration such as 0.6 mg mL^−1^, the optimum CNT content was 0.12 mg mL^−1^. The FTIR, XPS, and EDS results indicated that functionalization with the dendrimer occurred simultaneously with the reduction in the GO sheets, and the reduction degree and N content reached their limits at an optimum ratio of CNT.

The dendrimer-modified hybrid GO–CNT aerogels were applied for carbon capture at 25 °C. The CO_2_ uptake was found to increase with the increasing dendrimer content for the modified hybrid GO–CNT aerogels. The best performance in terms of CO_2_ sorption was exhibited by the aerogel modified with the PAMAM dendrimer at a high concentration value and optimum CNT content, reaching a value of 2.23 mmol g^−1^, in agreement with the reduction and functionalization degree. This performance is the highest reported value for dendrimer-modified GO aerogels thus far, and it is attributed to the addition of CNTs in the hybrid aerogels.

The results obtained in this work indicated that the adsorption performance of GO aerogels could be tailored by applying modifiers such as dendrimers for amine functionalization and by using CNTs as spacers, upon an appropriate balancing of their content. The approach followed in this work could be extended to other functionalization molecules as well as to other applications, such as those in sensing and electrochemical fields.

## Data Availability

Data available on request.
